# Mammalian Susceptibility to a Neonicotinoid Insecticide after Fetal and Early Postnatal Exposure

**DOI:** 10.1038/s41598-018-35129-5

**Published:** 2018-11-09

**Authors:** Andrew P. Burke, Yosuke Niibori, Hayato Terayama, Masatoshi Ito, Charlotte Pidgeon, Jason Arsenault, Pablo R. Camarero, Carolyn L. Cummins, Rafael Mateo, Kou Sakabe, David R. Hampson

**Affiliations:** 1 Department of Pharmaceutical Sciences, Leslie Dan Faculty of Pharmacy, University of Toronto, Toronto, Canada; 2 Department of Anatomy, Division of Basic Medicine, Tokai University School of Medicine, Kanagawa, 259-1193 Japan; 3 Support Center for Medical Research and Education, Tokai University School of Medicine, Kanagawa, 259-1193 Japan; 4 Instituto de Investigación en Recursos Cinegéticos (IREC) CSIC-UCLM-JCCM, Ronda de Toledo s/n, 13071 Ciudad Real, Spain; 5 Department of Pharmacology and Toxicology, Faculty of Medicine, University of Toronto, Toronto, Ontario, Canada

## Abstract

Neonicotinoids have become the most widely used class of insecticides world-wide. Although numerous studies have documented neonicotinoid toxicity in bees and other insects, the effects of exposure during early development in mammals remain largely unexplored. We assessed the effects of the neonicotinoid imidacloprid (IMI) in adult male and female mice after *in utero* and early postnatal exposure. Pregnant mice were infused with IMI (0.5 mg/kg/day) from gestational day 4 to the end of nursing at postnatal day 21. The young adult offspring were studied in a series of biochemical and behavioral tests. To assess reproducibility, the behavioral analyses were conducted in three separate studies using multiple exposed litters. Exposure to IMI reduced fecundity, and in adult offspring, decreased body weight in male but not female pups. Offspring from IMI-treated mothers displayed lower triglycerides, elevated motor activity, enhanced social dominance, reduced depressive-like behavior, and a diminution in social aggression compared to vehicle treated controls. Low levels of IMI were detected in the brains and livers of the treated mothers, while trace levels were detected in some offspring. Our results demonstrate that transient exposure to a neonicotinoid over the early developmental period induces long-lasting changes in behavior and brain function in mice.

## Introduction

Neonicotinoid pesticides are the most widely used and among the most effective insecticides on the planet^1–3^. Neonicotinoids were designed to be structurally similar to the potent toxin nicotine, and therefore act on central nervous system (CNS) nicotinic acetylcholine receptors (nAChRs; ref.^4^). While it has been widely assumed that neonicotinoids are selective for insect nAchRs with little or no activity toward mammalian receptors, the validity of this assumption has recently been challenged^5–7^. Thus, it becomes imperative to more rigorously investigate potential detrimental off-target effects of neonicotinoids on mammals including humans, livestock, and wildlife that may be exposed during pesticide application, or via contaminated food and water^8–12^.

Imidacloprid (IMI), a member of the chloronicotinyl nitroguanidine chemical family, was the first neonicotinoid to be commercialized and remains one of the most widely used insecticides^10^. It has been reported to act as a full agonist at house fly nAChRs^12^, or as a partial nAChR agonist in bees^13,14^, and chicken and Drosophila/chicken hybrid nAChRs expressed in *Xenopus laevis* oocytes^15^. In addition to structurally mimicking nicotine, IMI possesses the additional properties of being persistent in plants and soil and displaying high systemic toxicity against insects. An ideal insecticide is expected to quickly kill selected pests that harm crops while leaving other desirable species unaffected. However, several studies have reported off-target effects of IMI. For example, sublethal doses of IMI have been shown to cause neurotoxicity^16^, disrupt olfactory discrimination^17^, decrease sperm viability and fecundity, impair immunity, and alter responses to pheromones in bees and other insects^18–21^.

Studies conducted on adult mammals have shown that acute exposure to IMI at relatively high doses induces impaired immunity in both mice and rats^22,23^, genotoxicity in rabbits^24^, compromised reproductive function in rats (specifically, abnormal sperm morphology^25^), and oxidative stress and/or increased mortality in rats and increased mortality and reduced fat stores, body weight, and migratory orientation in birds^26,27^. In addition, IMI was reported to induce sensorimotor impairments in the offspring after single high toxic dose (337 mg/kg) in pregnant rats^28^. In separate groups of infant and adult rats, 2–8 mg/kg IMI given daily for 3 months impaired spatial learning and memory in the Morris water maze task^29^, a finding consistent with another study in rats using the neonicotinoid clothianidin^30^.

In light of the substantial body of knowledge detailing the detrimental effects of nicotine on the developing CNS following *in utero* and early postnatal exposure, and reports that neonicotinoids are present in fruits, vegetables, seeds, and honey^9,31–33^, it is essential to conduct more thorough testing to examine the developmental effects of neonicotinoids in mammals. The goal of the present study was to examine the effects of chronic *in utero* and early postnatal exposure of IMI in mice. After conception, pregnant mice were implanted with osmotic mini-pumps for slow infusion of IMI from gestational day 4 to the end of nursing at postnatal day 21. After reaching maturity the adult offspring of vehicle and IMI-exposed mothers were then subjected to a series of biochemical and behavioral tests to assess potential long-term alterations in motor and cognitive behaviors, and serum biochemical parameters. Our findings demonstrate the induction of abnormal behaviours in mice after *in utero* and early postnatal exposure to IMI.

## Experimental Procedures

### Study design

The selection of behavioral tests employed was based on endophenotypes that have been shown to be affected by nicotine. This series of experiments was conducted serially over 18 months on three separate groups of mice; the three groups were designated Study A, B, and C. To assess reproducibility, several of the tests were repeated in two, or in all three studies (see Supplementary Table 1 for a summary of the tests conducted in each of the three studies). The data were analyzed initially via Two-way ANOVA with treatment group and sex constituting the two arms. When no significant sex differences were observed, male and female data were combined for assessment of the treatment effect. We note that ovariectomies were not performed and estrous cycles were not synchronized; this may have influenced the results of some tests in female mice. Additional groups of mice treated with either IMI or vehicle (DMSO) were generated for the collection of serum, and for IMI tissue quantitation.

### Animals and Treatments

All animal experiments were carried out in accordance with the guidelines set by the Canadian Council on Animal Care and were approved by the University of Toronto Animal Care Committee. All mice were fed Teklad Global 18% protein rodent diet (#2918, Envigo). All mice were kept in standard mouse cages housed 2–4 mice per cage, given food and water ad libitum, and maintained at 22–23 °C on a 12-hour light/dark cycle. Adult (7–10 weeks old) female CD-1 mice (Charles River Inc.) were mated with male CD-1 mice. The status of the vaginal plug was assessed each day at 9 a.m. and 4 p.m.; the day the vaginal plug was present was designated as gestational day 0. For solubility testing, preliminary experimentation using a range of vehicle (dimethyl sulfoxide, DMSO) concentrations established that at DMSO concentrations of less than 15%, IMI precipitated out of solution; therefore, 25% DMSO was used to ensure IMI would remain in solution during chronic treatment.

Between gestational days 3–6, pregnant female mice were implanted with an osmotic mini-pump (Alzet Osmotic Pumps: Model 2006, 200 µL reservoir, 0.15 µL/hour flow rate, 42 days-duration) dispensing 0.5 mg/kg/day of IMI (PESTANAL 37894, Sigma-Aldrich) dissolved in 25% DMSO, or 25% DMSO alone for vehicle controls. For treatment via mini-pump, pregnant females were randomly allocated to either the vehicle or IMI exposure groups. In total, 10 pregnant females were implanted with mini-pumps containing the DMSO vehicle and 10 were implanted with IMI dissolved in DMSO. The offspring were housed with their mother until weaning on postnatal day (PND) 21. After weaning the offspring were sex-matched and housed with siblings in groups of 2–4 per cage. The mice were left undisturbed, with the exception of a biweekly cage cleaning until behavioural testing was initiated. All testing was performed blind and carried out between 9 a.m. and 5 p.m. beginning on PND 42. Each mouse was naïve to each test and was subjected to each test only once. Behavioural analyses were arranged such that all mice participated in a maximum of five tests; the less stressful tests were performed at the beginning (e.g. open field test) or earlier in testing series, while the more stressful tests (e.g. the forced swim test) were performed later in the testing regimen.

### Open Field Test

The open field test measuring general motor activity was performed on PND 43–47 between 1 p.m. and 5 p.m. The basic procedures have been described previously^34,35^. Briefly, on the day of testing, mice were acclimated to the testing room which was dimly lit (~40 lux) by two 20 W light fixtures for 15 min. The open field test was performed using an automated open field monitoring system (Omnitech Electronics, Inc.). An automated monitoring system consisted of a 42 × 42 × 30 cm plexiglass box was placed inside a detection system, which emitted 16 × 16 × 16 laser beams, each separated by 2.5 cm. Movement was tracked by the animal disrupting the laser beams and the data were automatically quantitated by the system software. The test began with a mouse being placed in the center of the chamber, free to explore for one 20-min. trial. At the conclusion of the trial, the mouse was returned to its home cages and all materials and testing surfaces were cleaned. The time spent in the center zone and total distance travelled was recorded. The data obtained from the trial was analyzed in one 20-min. bin and in two 10-min. bins. A two-way ANOVA and a Bonferroni post-hoc test were used for statistical analysis. When statistically justified, males and females were combined, and DMSO and IMI treatment groups were compared using a two-tailed Student’s t-test with a 95% confidence interval.

### Elevated plus maze

The elevated plus maze test measuring anxiety was conducted on PND 47–54 between 9 a.m. and 1 p.m. On the day of testing, the mice were acclimated to the testing room (with normal lighting) for 15 min. The apparatus consisted of two opposing open arms, two opposing closed arms, and a center zone; open arms are adjacent to closed arms, and all arms are at a 90° angle to each other. The mouse was placed in the center zone, facing an open arm and was left to explore the maze for one 5-min. trial. During the test, video recording software (Viewer^2^ BIOBSERVE GmbH) recorded the animal’s movements and position. The time spent in the open arms, closed arms, and center zone was recorded and analyzed. Two-way ANOVA with Bonferroni and Tukey post-hoc tests were used for data analysis.

### Forced swim test

The forced swim test measuring depressive-like behavior was conducted on PND 61–67 between 9 a.m. and 1 p.m. The mice were acclimated to the testing room for 30 minutes (dimly lit ~40 lux). The testing session was video recorded and was initiated by placing a mouse, tail first, into a 2000 mL beaker filled with 1600 mL of tap water (22–24 °C). The mouse was free to swim in the beaker for one 6-min. trial. At the conclusion of the 6-min. trial, the mice were dried with a towel under a heat lamp, and returned back to their home caging. The water was replaced and the beaker was cleaned when it became visibly soiled. The final 4 min. of the 6-min. video recording were analyzed by measuring the time spent swimming and the time spent immobilized. Two-way ANOVA with Bonferroni post-hoc analysis was used for statistical computation. In cases where no significant sex effect was detected, males and females were combined and DMSO and IMI treatment groups were compared using a two-tailed Student’s t-test with a 95% confidence interval.

### Tube test

The tube test to assess social dominance took place on PND 54–64 between 1 p.m. and 5 p.m. On the day of testing, the mice were individually caged and acclimated to the testing. Before each trial, all materials and testing surfaces were cleaned with Virox followed by water. The tube test consisted of placing two sex-matched and age-matched mice, from two different treatment groups, at opposite ends of a transparent polyvinyl chloride tube (inner diameter: 2.5 cm, length: 30.5 cm). The mice were released simultaneously with both mice entering the tube. The test concluded when one mouse placed at least two hind paws outside the tube, with the mouse remaining inside the tube being declared the dominant winner; subsequently both mice were returned to their individual cage, and the tube was cleaned with Virox followed by water. This procedure was repeated a maximum number of times (5 matchups per mouse), such that the same matchup was never repeated. At the conclusion of the test, all mice were returned to their original home caging, and all materials and testing surfaces were thoroughly cleaned. The number of wins and winning percentage per treatment group were compared. The data were analyzed using Fisher’s Exact Test for statistical significance.

### Resident intruder test

The resident intruder test was used to examine the effects of IMI on social aggression and dominance. The test was conducted on male mice on PND 66–72 between 1 p.m. and 5 p.m. Eleven to seventeen days prior to testing (PND 55–59), the mice were individually caged. On the day of testing, the mice were acclimated to the testing room (normally lit) for 20 min. A video camera recorded the activity of the mice during the test. The test began with the resident cage being placed in front of the video camera, and the age-matched and sex-matched wild type CD-1 intruder mouse being placed in the resident cage. The resident and intruder mice were free to interact for 10 min. At the conclusion of the test, the intruder mouse was returned to its home cage. The number of attacks (count), the duration of attacks (measured in seconds), and the total fight time (in seconds) were recorded for both the resident and the intruder. In addition, the latency to the first attack (measured in seconds) was also recorded, for the resident or the intruder (whichever initiated the first attack). Total fight time (in seconds) was calculated by adding the duration of attacks by resident to the duration of attacks by intruder. A two-tailed Student’s t-test with a 95% confidence interval was used as a test for statistical significance.

### Serum biochemistry

Serum was prepared from whole blood collected from young adult female offspring via cardiac puncture. Biochemical analysis of serum samples was conducted at the Toronto Centre for Mouse PhenoGenomics at the Hospital for Sick Children in Toronto using a Beckman Coulter AU480 analyzer.

### Confirmation of dosing and tissue residues

Tissue samples from implanted mothers were collected 38–44 days after initial pump implantation, while tissue samples from the offspring were collected 60–70 days after initial pump implantation in the mother. Two LC/MS methods were used for quantitation of IMI in tissues from both the implanted mothers and offspring mice. In the first, samples of whole brain and liver were dissected from DMSO or IMI-treated young adult offspring and frozen at −80 °C. Samples were thawed, weighed, and extracted twice with acetonitrile at 4 °C using a Sonicator 3000 (Misonix Inc.) tissue disruptor for 2 min. followed by vigorous vortexing for 2 min. After centrifugation at 2000 × g for 15 min., the combined liquid extracts were evaporated to dryness under N_2_ at 25 °C. For quantitation of IMI, the samples were reconstituted in methanol and filtered through a 0.2 μm nylon filter syringe. Electrospray ionization LC/MS analysis was carried out using an LCMS-8050 triple quadrupole mass spectrometer (Shimadzu, Kyoto, Japan) with a LC-30A Nexera UHPLC system (Shimadzu, Kyoto, Japan). All analyses were conducted blind. The analysis was performed on an L-column2 ODS (2.1 mm × 150 mm, 3 mm; Chemicals Evaluation and Research Institute (CERI), Tokyo, Japan) at 40 °C. Gradient elution was used with solvents consisting of 0.05%(v/v) formic acid, 0.1%(v/v) acetic acid in ultra-pure water (solvent A) and methanol (solvent B). The elution gradient was 2% B to 95% B (2–10 min), 95% B (10–12 min), and 2% B (12–15 min) at a flow rate of 0.3 ml/min.

A triple quadrupole LCMS-8050 (Shimadzu) equipped with an ESI interface operating in the positive mode was used. Imidacloprid and Imidacloprid-D_4_ (deuterated IMI, added at the beginning of the extraction procedure) were detected by multiple reaction monitoring (MRM) in the positive ion mode, the ion transition m/z 256.1 → 175.2 and 209.15 for IMI and 260.1 → 213.1 and 179.15 for IMI-D_4_ were used. The following ESI inlet conditions were used. Nitrogen was used as a nebulizer gas with a flow rate of 3 l/min, the ESI probe temperature was 300 °C, the CDL temperature was 250 °C, and the detector voltage was 2.28 V. The peak-areas for all components were automatically integrated using Labsolutions Version 5.86 software (Shimadzu Chemical Laboratory Analysis System & Software, Kyoto, Japan).

For the second method used to assess IMI levels in DMSO and IMI-treated mothers, quantitation was performed as described in López-Antia *et al*.^26^ with some modifications. Samples (60–270 mg) were homogenized in 2 ml of acetonitrile in a homogenizer WISD WiseTis® after adding 20 µl of internal standard (thiacloprid) at a concentration of 1 ng/µl. Homogenate was centrifuged at 10,000 × g for 5 min. and filtered through a 2 µm de Nylon filter syringe. The analysis was performed with a HPLC system (Shimadzu LC30AD) coupled through an DuoSpray Ion Source (TurboIon Spay and APCI) to a quadrupole-time-of-flight (QTOF) mass spectrometer (TripleTOF 4600-1, AB Sciex Instruments). The temperature of TurboIon Spray was set at 500 °C. The injection volume was 4 µl. The chromatographic method was as described in López-Antia *et al*. 2013 (ref.^26^) but using acetonitrile instead of methanol as organic solvent. The chromatographic separation was performed on reverse-phase column (Poroshell 120 2.7 μm EC-C18, 150 mm × 2.1 mm i.d.). The mass spectrometer operated in the positive ionization mode, with an ion spray voltage floating of 5500 V, a flow rate of curtain gas (N_2_) of 9 L/min at 500 °C, and ion source gas GS (N_2_) flow of 30 L/min. Spectra were acquired in a mass range of mass/charge (m/z) of 100–2000. The m/z scale of the mass spectra was calibrated every 15 samples with a commercial calibration solution for APCI. MS/MS fragmentation used to identify IMI in samples was acquired from ions 256.06 m/z and 253.03 m/z as precursors of IMI and thiacloprid as internal standard respectively. The quantification was performed with the product ion 209.06 m/z for IMI and 198.93 m/z for thiacloprid. The compound parameters of mass were a collision energy 35 eV and declusting potential of 50 eV. Recorded data were processed with software Peak View 2.2 The LOD and LOQ calculated based on the SD of the response and the slope for 0.2 g of sample were 0.11 ng/g and 0.33 ng/g, respectively.

### nAchR autoradiography

nAchRs were measured in control and IMI-treated brains from the offspring of the treated mothers using the nAChR ligand [^3^H]epibatidine (Perkin Elmer Corp. product #NET 1102; specific activity, 54 Ci/mmol) and a modified version of the protocol described by Whiteaker *et al*.,^36^ was conducted as follows. Sections (14 uM thickness) from unfixed frozen brains were cut on a cryostat, mounted on gelatin-coated slides, and dried. Sections were rehydrated in binding buffer (144 mM NaCl, 1.5 mM KCl, 2 mM CaCl_2_, 1 mM MgSO_4_, 20 mM HEPES, pH 7.5) at 22 °C for 15 min., followed by incubation with 1 nM [^3^H]epibatidine in binding buffer for 70 min. at 22 °C. (-) Nicotine (300 uM; ditartrate, Tocris) was used to define non-specific binding which was subtracted from total binding to obtain specific [^3^H]epibatidine binding. The slides were then washed once in binding buffer (at 4 °C) for 1 min. followed by drying under a stream of air for 48 h, after which they were apposed to Kodak Biomax MR film (Carestream Inc.) for 12 weeks at room temperature. The film was developed manually according to the manufacturer’s instructions. For quantitation, the positions of secondary motor cortex, superior colliculus superficial grey, thalamus and interpeduncular nucleus were determined using the Mouse Brain in Stereotaxic Coordinates (Academic Press). The intensity of the selected brain regions from 2 to 3 brain slices per mouse were quantified, and the average intensity of the blanks incubated with (-) nicotine were subtracted from each.

## Results

### Fecundity, body weight, and serum biochemistry

**Figure 1:**
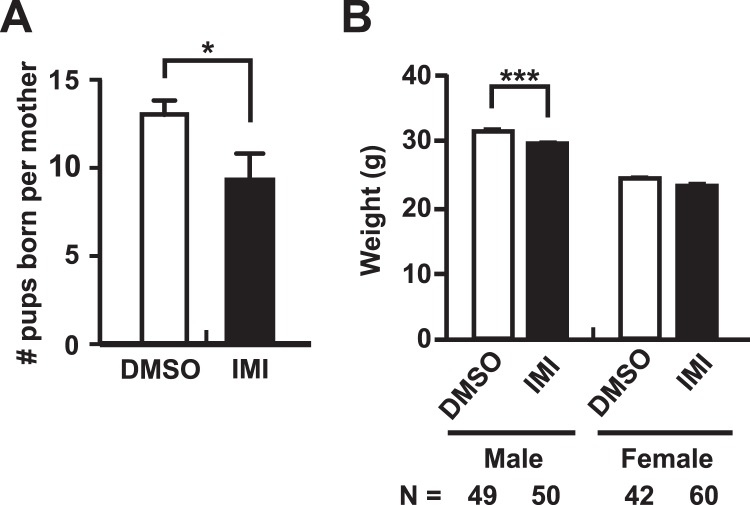
Summary of fecundity and body weight analysis. (**A**) Summary of fecundity. (**B**) Body weight analysis of mice. Mice were weighed between PND 42–46. In both panels each column represents the mean ± SEM; N = number of mice in each group. Student’s t-test was used for the data in panel A, while two-way ANOVA with Bonferroni Post-hoc was used for data in panel B. *p < 0.01; ***p < 0.001.

Observation of all pregnant females after implantation revealed no mortality or obvious signs of toxicity or overt distress in either treatment group. Fecundity was defined as the number of offspring that were born and survived for more than one week. The number of mice born from IMI-treated CD-1 mouse mothers was lower than that from control DMSO-treated mothers (Fig. 1A). The number of males vs. the number of females born did not differ between the two treatment groups.

Body weights were recorded in Studies B and C between postnatal day (PND) 42–46 as a static measure of overall health. Analysis by two-way ANOVA indicated both a significant treatment effect in Study B (F = 29.46, p < 0.0001) and in Studies B and C combined (F = 18.28, p < 0.0001), and a significant sex effect in Study B (F = 221.9, p < 0.0001), Study C (F = 244.9, p < 0.0001) and in Studies B and C combined (F = 396.9, p < 0.0001); therefore, further statistical analyses were carried out separately on male and female body weights. In Study B, a significant decrease in body weight in the IMI treatment group compared to the DMSO control treatment group was observed in both males (p < 0.0001) and females (p < 0.01). In Study C the IMI treated male mice weighed less than control males but the difference was not significant for either sex. When the data from Study B and Study C were combined, a significant decrease in body weight in the IMI treatment group was observed in males (P < 0.001) but not females (p > 0.05; Fig. 1B).

Samples of serum were collected from young adult offspring of DMSO and IMI-treated mothers and analyzed for 18 biochemical parameters (Table 1). Although glucose and alkaline phosphatase were reduced, this did not reach statistical significance. However, triglycerides were highly and significantly reduced in IMI (114.5 mg/dL) mice compared to DMSO-treated mice (197.8 mg/dL; p < 0.005).

**Table 1:** Summary of serum biochemistry analysis. Each value is the mean ± S.E.M.

	DMSO	IMI	p value (DMSO vs IMI)
ALT	53.1 ± 8.8	44.5 ± 3.7	0.3854
AST	293.4 ± 30.9	280.9 ± 33.6	0.7867
ALP	101.8 ± 7.5	83.9 ± 5.4	0.0679
TBIL	0.2 ± 0	0.2 ± 0.0	0.9410
TP	47.8 ± 0.7	46.6 ± 0.7	0.2640
ALB	28.1 ± 0.5	27.9 ± 0.4	0.7745
CHOL	120.1 ± 4	123.2 ± 8.0	0.7319
HDL	59.6 ± 2.4	58.8 ± 4.5	0.8889
LDL	4.6 ± 0.3	4.9 ± 0.4	0.4704
TRIG	197.8 ± 22.0	114.5 ± 9.8	0.0039***
PHOS	11.1 ± 0.4	11.1 ± 0.4	0.9200
CAL	10.8 ± 0.2	10.4 ± 0.1	0.0965
GLU	362.8 ± 16.3	318.3 ± 15.4	0.0598
CRE	0.1 ± 0.0	0.2 ± 0.0	0.0809
BUN	24.6 ± 1.1	24.4 ± 0.7	0.9140
Na	142.6 ± 0.6	143.4 ± 1.1	0.5633
K	5.9 ± 0.2	5.7 ± 0.2	0.4956
Cl	109.4 ± 0.5	109.5 ± 0.9	0.9203

N = 11 for DMSO samples and N = 14 for IMI samples. ***p < 0.005. Abbreviation note: LT = Alanine amminotransferase (IU/L); AST = Aspartate aminotransferase (IU/L); ALP = Alkaline phosphatase(IU/L); TBIL = Total bilirubin (mg/dL); TP = Total protein (g/L); ALB = Albumin (g/L); CHOL = Total cholesterol (mg/dL); HDL = HDL Cholesterol (mg/dL); TRIG = Triglycerides (mg/dL); PHOS = Phosphorus (mg/dL); CAL-Calcium (mg/dL); GLU = Glucose (mg/dL); CRE = Creatinine (mg/dL); BUN = Blood urea nitrogen (mg/dL); Na = Sodium(mmol/L); K = Potassium(mmol/L); Cl = Chloride (mmol/L).

### Open field test and elevated plus maze

**Figure 2:**
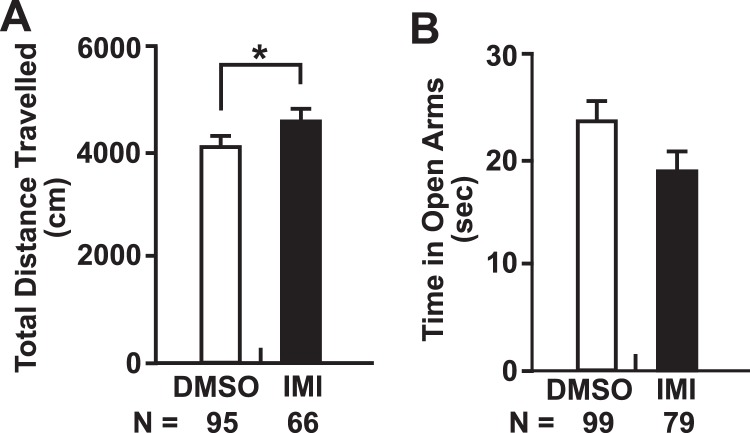
Summary of open field testing and elevated plus maze. (**A**) Compilation of motor activity from combined males and females in Study A + Study B; (**B**) Compilation of open arm analyses from the elevated plus maze from Studies A + B. Each column represents the mean ± SEM. N = number of mice in each treatment group. Two-way ANOVA with Bonferroni post-hoc test was used for data in panel A, and a two-tailed Student’s t-test in was used in the data shown in panel B. *p < 0.05.

The open field test was used to analyze motor activity (reflected in the total distance travelled) of the adult offspring in Studies A and B. Two-way ANOVA indicated a significant sex effect in Study B (F = 4.974, P = 0.0281) but not in Study A. Therefore, in Study B only, males and females were analyzed separately using two-way ANOVA, while in Study A and in Study A and B combined, the males and females were pooled for subsequent statistical analysis using the two-tailed Student’s t-test. In Study A, no significant difference in the total distance travelled by the DMSO and IMI treatment groups was observed (Suppl. Fig. 1A). In Study B, a non-significant trend of increased total distance travelled in the males of the IMI treatment group was observed, compared to the DMSO treatment group (P > 0.05; Suppl. Fig. 1B). Pooled data from both males and females from Study A and Study B together showed a significant increase in total distance travelled observed in the IMI-treated mice compared to the DMSO treatment group (P < 0.05; Fig. 2A).

The elevated plus maze, a well-established test used to assess anxiety and exploratory behavior^35^, was employed in Studies A and B. Reduced time spent in the open arms of the maze indicate increased anxiety. Two-way ANOVA analysis indicated no significant treatment effect in Study A (F = 1.658, p = 0.2018), in Study B (F = 1.909, p = 0.1703), or in Study A and Study B combined (F = 2.701, p = 0.1021), and no significant sex effect in Study A (F = 0.3279, p = 0.5686), Study B (F = 0.6828, p = 0.4107), or in Study A and Study B combined (F = 0.09784, p = 0.7548). Therefore, the results from males and females were combined and a two-tailed Student’s t-test was used to assess the differences between IMI and DMSO. Although there was a trend towards reduced time in the open arms in Study A (Suppl. Fig. 1C) and in Study B (Suppl. Fig. 1D), and in the combined data set (Fig. 2B), no significant differences in the time spent in the open arms were observed between the IMI and DMSO treatment groups.

### Forced swim test

The forced swim test is a test of behavioural despair and is commonly used to assess depressive-like behavior. The results of a two-way ANOVA analysis indicated both a significant treatment effect in Study A (F = 10.91, p = 0.0015), Study B (F = 13.92, p = 0.0004), and in Studies A and B combined (F = 37.26, p < 0.0001), and a significant sex effect in Study A (F = 6.360, p = 0.0138), Study B (F = 13.73, p = 0.0004), and Studies A and B combined (F = 24.58, p < 0.0001). In addition, an interaction between treatment and sex was present when Studies A and B were combined (F = 6.003, p = 0.0155). Therefore, further statistical analyses were carried out on male and female data separately.

**Figure 3:**
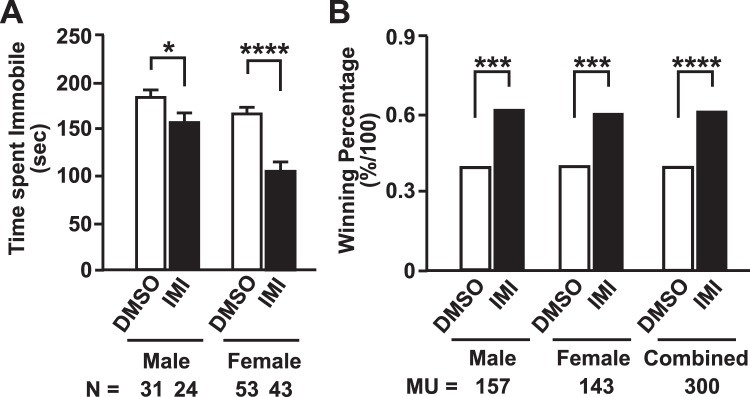
Summary of forced swim test measuring depressive-like behavior and the tube test measuring social dominance. (**A**) Compilation of the forced swim test results from Studies A + B. Each column represents the mean ± S.E.M. N = number of mice in each group. Two-way ANOVA with Bonferroni Post-hoc test. *p < 0.05; ****p < 0.0001. (**B**) Compilation of tube test analyses from Studies A, B, and C. No mice were used in more than 5 matchups. The data are presented as the winning percentage, calculated as the number of wins divided by the total number of matchups. MU = number of matchups between the treatment groups. Fisher’s exact test, ***p < 0.001, ****p < 0.0001.

In Study A, a significant decrease in the time spent immobile in the IMI treatment group females (p < 0.01) compared to the DMSO treatment group was observed (Suppl. Fig. 2). In Study B, a significant decrease in the time spent immobile was again observed in the female IMI treatment group compared to the DMSO treatment group was observed (p < 0.0001; Suppl. Fig. 2B). When the results from Study A and Study B were combined a significant reduction in the time spent immobile was observed in the IMI treatment groups for both males (p < 0.05) and females (p < 0.0001) compared to the corresponding DMSO treatment groups (Fig. 3A). These results indicate a reduction in depressive-like behavior in the offspring of IMI-treated mothers.

### Tube test

The tube test is used to evaluate social dominance^34,35^. In Study A, IMI-treated offspring were tested against DMSO offspring in both males and females combined; no significant difference between the treatment groups was observed (Suppl. Fig. 3A). However, in males, a significant increase in winning in the IMI treatment group was observed (p < 0.05) compared to the DMSO treatment group (Suppl. Fig. 3A). In the females, a significant decrease in winning in the IMI treatment group was observed (p < 0.05), compared to the DMSO treatment group (Fig. 3A).

In Study B, when DMSO-treated mice were matched against IMI mice, a significant increase in winning in the IMI treatment group was observed in the males (p < 0.001), females (p < 0.001), and males and females combined (p < 0.001), compared the DMSO treatment group Suppl. Fig. 3B). In Study C, the females alone, and the females and males combined, showed a non-significant trend towards more wins in the IMI-treated mice (Suppl. Fig. 3C). When test results from Studies A, B and C were combined, the winning percentage in the IMI treatment group was significantly increased in the males alone (p < 0.001), in the females alone (p < 0.001), and in the males and females combined (p < 0.0001), compared to the DMSO treatment group (Fig. 3B). Thus, the results of the tube test indicated that IMI treatment induces increased social dominance behavior.

### Resident intruder test

The resident intruder test was conducted on male mice in Studies B and C to further probe the effects of IMI on social aggression and dominance. In Study B, a significant decrease in the number of attacks by the IMI treatment group residents was observed (p < 0.05), compared to the DMSO treatment group (Suppl. Fig. 4A). In addition, the duration of the attacks by the resident mouse was significantly decreased in the IMI treatment group residents (p < 0.05) (Suppl. Fig. 4B), and a significant decrease in total fight time was also observed in the IMI treatment group compared to the DMSO treatment group (p < 0.05; Suppl. Fig. 4B). The numbers of attacks by the intruder, and the duration of the attacks by the intruder, were decreased in the IMI treatment group compared to the DMSO treatment group, but this parameter was not statistically significant (p > 0.05, Suppl. Fig. 4D,E).

In the Study C resident intruder test analysis, the attacks by resident, the duration of attacks by resident, and the total fight time, were decreased in the IMI treatment group residents, compared to the DMSO treatment group, but these parameters were not statistically significant (P > 0.05; Suppl. Fig. 4F–H). In addition, no significant differences in the number of attacks by intruder, or duration of attacks by intruder, were observed between treatment groups (P > 0.05; Suppl. Fig. 4I,J).

**Figure 4:**
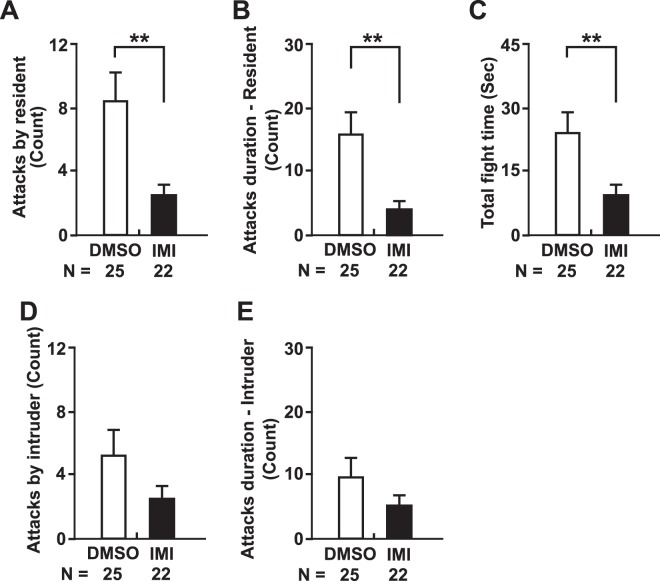
Resident intruder test of aggression. All tests were conducted using male mice. (**A**) Attacks perpetrated by the resident mouse, (**B**) Mean resident attack duration, (**C**) Total fight time between resident and intruder, (**D**) Attacks by intruder on resident mouse, (**E**) Mean intruder attack duration. Each column represents the mean ± SEM. N = number of mice in each group. Two-tailed Student’s t-test; **p < 0.01.

When the results from Studies B and C were combined, a significant decrease in the number of attacks by the IMI treatment group residents was observed (p < 0.01) compared to the DMSO treatment group (Fig. 4A), and the duration of the attacks by the resident was decreased in the IMI treatment group residents compared to the DMSO treatment group (p < 0.01; Fig. 4B). Additionally, a decrease in total fight time was also observed in the IMI treatment group (p < 0.01), compared to the DMSO treatment group (Fig. 4C). No significant differences in the number of attacks by intruder, or duration of attacks by intruder were observed between treatment groups (p > 0.05; Fig. 4D,E). These results demonstrate that interactive mouse-to-mouse aggression was highly reduced in offspring from IMI-treated mothers compared to offspring from vehicle-treated mothers.

### Quantitation of IMI in tissues

**Figure 5:**
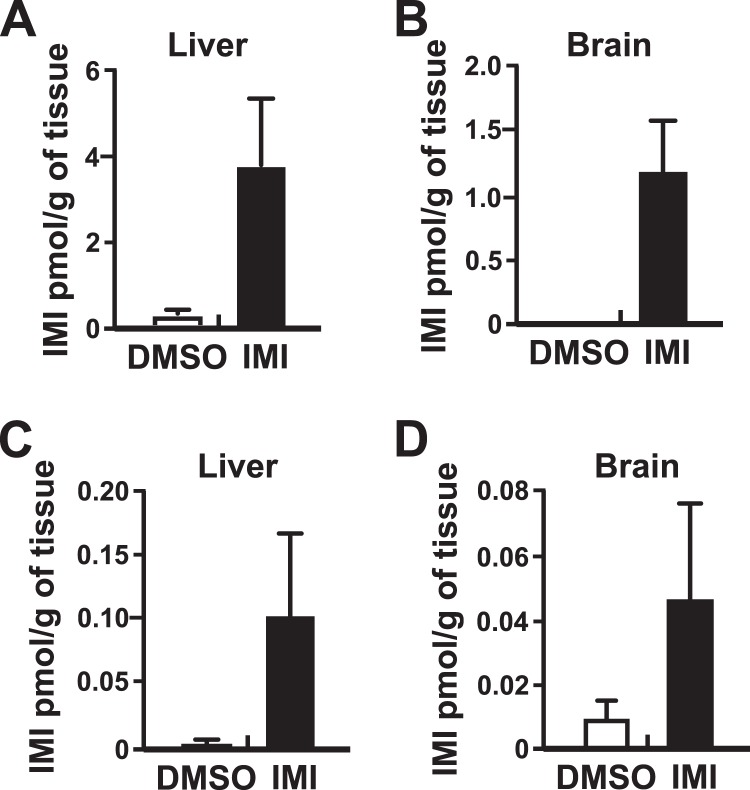
Quantitation of IMI levels in liver and brain. Tissues from DMSO and IMI-treated mothers and their offspring were collected, extracted, and analyzed by LC/MS. (**A**) IMI levels (mean ± S.E.M.) in livers from implanted mothers: DMSO, 0.185 ± 0.185 pmol/g tissue, N = 4; IMI, 3.71 ± 1.54 pmol/gram tissue, N = 6. (**B**) IMI levels in brain tissue from mothers: DMSO, not detected, N = 4; IMI, 1.18, ± 0.413 pmol/gram tissue, N = 6. (**C**) IMI levels in livers of young adult offspring; DMSO, 0.0044 ± 0.0044 pmol/gram, N = 4; and IMI treated mice, 0.104 ± 0.064 pmol/gram, N = 12. (**D**) IMI levels in brains of young adult offspring; DMSO treated, 0.0075 ± 0.0047 pmol/gram, N = 4; and 0.044 ± 0.0291 pmol/gram tissue, N = 13 for IMI-treated mice.

To determine whether chronic exposure to IMI via an osmotic mini-pump implanted subcutaneously resulted in detectable levels of IMI in tissues, the livers and brains from treated mothers and their offspring were collected and analyzed using LC/MS (Fig. 5). Samples of liver and brain collected from mothers and offspring from mothers treated with the DMSO vehicle had levels of IMI that were either below the limit of detection, or in a few mice, slightly above the limit of detection, possibly as a result of low level contamination in the mouse chow. In the IMI-treated mothers, IMI was variably detected in tissues of some mice with higher levels observed in liver compared to brain (Fig. 5A and B). As expected, the levels in the treated mothers (Fig. 5A,B) were higher than those in the exposed offspring particularly in the livers of offspring mice which displayed highly variable levels of IMI (Fig. 5C and D).

### nAchR autoradiography

**Figure 6:**
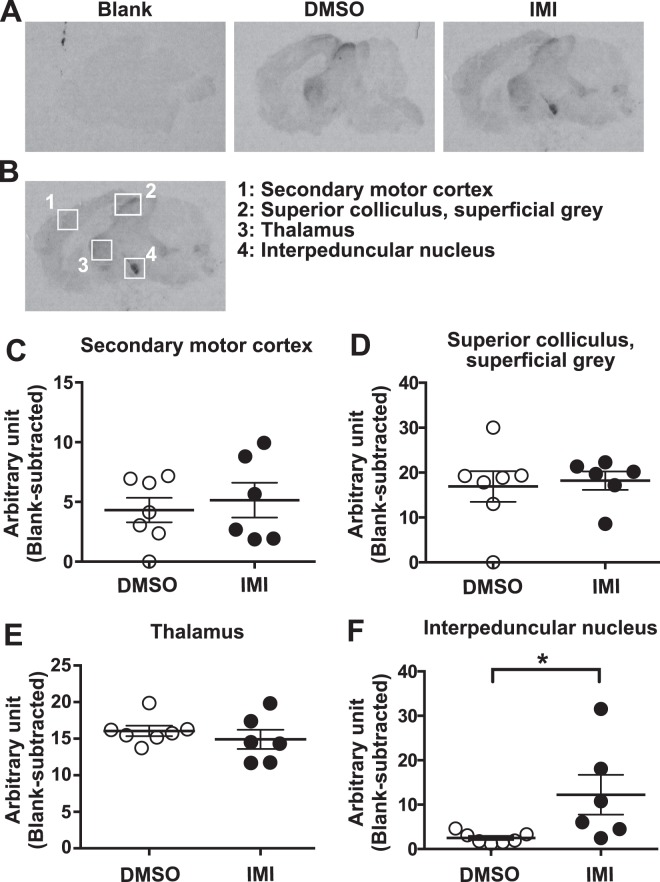
nAChR autoradiography using [^3^H]epibatidine. Sections of brain tissue from vehicle controls and IMI-treated offspring mice were prepared and exposed to the nAChR ligand [^3^H]epibatidine. (**A**) Representative sagittal brain sections showing blank (incubated with [^3^H]epibatidine plus unlabelled nicotine to block non-specific binding sites), DMSO-treated, and IMI-treated mice. (**B**) Locations of regions sampled in panels C-F. (**C**–**F**) Specific nAChR receptor binding was quantified in selected brain regions including the thalamus, cerebral cortex, superior colliculus, and interpeduncular nucleus in DMSO-treated mice (N = 7) and IMI-treated mice (N = 6). *P < 0.05.

To assess the status of nAChRs in the CNS after chronic IMI exposure (via osmotic mini-pump as described above), sections of brain from treated offspring mice were incubated with [^3^H]epibatidine (Fig. 6A) and receptor binding was quantified in selected brain regions (Fig. 6B–F). [^3^H]epibatidine was not different in IMI-treated vs. DMSO-treated mice in the thalamus, cerebral cortex, or superior colliculus; however, binding in the interpeduncular nucleus, a region known to contain a high density of nAChRs^36–38^, was significantly increased in IMI-treated mouse brains (Fig. 6F).

## Discussion

Unlike prenatal exposure to nicotine which has been examined in detail in both rodents and humans, the effects of prenatal and early postnatal exposure of mammals to neonicotinoids has remained relatively unexplored. We sought to begin to fill this gap by investigating the developmental effects of IMI, one of the most widely used neonicotinoids. We administered IMI to pregnant female mice during gestation and during the nursing period. Mice were exposed to 0.5 mg/kg/day of IMI from the first gestational week until approximately the time of weaning at PND 21. Based on the manufacturer’s specifications, the implanted mini-pumps were expected to be functional for 42 days, thereby theoretically covering the entire period from implantation up to weaning on PND 21. Behavioral testing commenced with an analysis of motor activity between PND 43–47, which was 22–26 days after weaning. We made the assumption that IMI exposure continued after birth via ingestion of the mother’s milk. Although no studies have investigated the presence of IMI in mother’s milk after subcutaneous or oral exposure, other neonicotinoids such as acetamiprid have been shown to cross the blood-brain barrier in mice after oral ingestion and enter and accumulate in the CNS^39,40^. Penetration into the CNS has also been demonstrated in bumblebees where exposure to 10 nM (2.1 ppb) IMI in the diet translated into accumulation of 4–10 nM IMI within the brain after 3 days of treatment^41^.

The 0.5 mg/kg/day dose of IMI used here is lower than that in almost all prior animal studies of this neonicotinoid. This dose, although likely to be higher than typical environmental exposure levels, represents a reasonable starting point for assessing the developmental effects of IMI in mammals. While the concentrations of neonicotinoids to which humans are exposed to has not been thoroughly established, studies quantitating neonicotinoids in human urine have reported a wide range of values^42–45^. In humans, IMI is metabolized in the liver to 6-chloronicotinic acid, 5-hydroxy-imidacloprid, 4,5-dihydroxy-imidacloprid and other metabolites^43,44^, while about 10–20% is excreted unchanged in the urine over several days subsequent to exposure^45^. However, it is difficult to compare human urine levels with our results in organ tissues. While the dose used in our mouse study was low relative to those used in animal studies published to date, it is likely higher than the levels of neonicotinoids that humans may be exposed to from ingesting contaminated food and water. Nevertheless, future animal studies should seek to determine the lowest dose of IMI and other neonicotinoids that induce biochemical and behavioral abnormalities, and concomitantly quantitate tissue levels (including urinary excretion). Another useful endeavor would be to assess neonicotinoid levels in human postmortem tissues which could then be compared with results from animal studies where the dose and length of exposure are known.

Results obtained from the LC/MS quantitation of IMI in mouse tissues illustrate the ability of peripherally administered IMI to accumulate in the livers and brains of the treated mothers. Moreover, the presence of trace levels of IMI detected in the brains and livers of some of the offspring 3–4 weeks after the end of exposure at weaning, indicate that IMI can be transferred from the mother to the pups; exposure of the mouse pups likely occurred through the fetal blood circulation and via mother’s milk. The inability to detect IMI in all treated animals could be explained by metabolism and elimination of the insecticide prior to sample collection, and in the case of the offspring, the long delay in samples collection (60–70 days after pump implantation in the mother), which corresponded to the time of completion of the behavioral testing.

As noted, most previous studies of neonicotinoid exposure in rodents used doses that were higher than the 0.5 mg/kg dose used in the present study, assessed acute effects of single doses, and/or did not administer the insecticide during both the prenatal and early postnatal period. In terms of experimental design, the study most similar to the present study was conducted by Kara *et al*.^29^. This group administered IMI to infant and adult rats at 2–8 mg/kg daily for 3 months via the oral route, and conducted a single behavioral test, the Morris water maze where the treated rats displayed impaired spatial learning and memory. We observed that transient exposure of CD-1 mice to IMI induced weight loss in male mice but not female mice, a mild elevation in motor hyperactivity, reduced depressive-like behavior, enhanced social dominance in the tube test, and decreased aggressiveness in the resident intruder test. Although the implanted mothers did not undergo behavioral testing, no overt abnormal or “sickness” behaviors were observed in any of the implanted females. Although unlikely, we cannot rule out the possibility that the mothers displayed subtle abnormal behaviors that influenced the behaviors of their pups.

Decreased body weight and elevated motor activity in IMI-treated male mice is consistent with the findings with nicotine^46^, and with the neonicotinoids dinotefuran and clothianidin where exposure during the prenatal and early postnatal periods also induced increased motor activity in adult mice^47,48^. Reduced body weight in the male IMI-treated mice is consistent with observations demonstrating that cholinergic neurons in the basal forebrain potently influence food intake and body weight. For example, impaired cholinergic signaling in mice increased food intake and induced severe obesity, whereas enhanced cholinergic signaling decreases food consumption and body weight^49^. In this context, our findings suggest elevated nAChR signaling after chronic neonatal IMI exposure.

The results of the forced swim test and the resident intruder test also revealed significant changes compared to vehicle-treated mice. The decrease in immobility in the forced swim test suggests reduced “depressive-like behavior”. This outcome is compatible with previous studies conducted on nicotine-treated rats^50^ and humans^51^ showing anti-depressant like behaviors after chronic nicotine exposure (see ref.^52^ for review). It is conceivable that decreased immobility in IMI-treated mice might have been due, at least in part, to the reduced body weight and/or mildly elevated motor activity whereby the slightly leaner IMI-treated mice may have had to keep moving continuously to keep their heads above the water line. However, arguing against body weight as a factor is the observation that the reduced body weight was only significant in the male mice, while in the forced swim test, both the consistency and magnitude of the decreased immobility was greater in the female mice.

The observations that IMI-treated mice showed increased social dominance in the tube test, while also demonstrating reduced mouse-to-mouse confrontation in the resident intruder test, at first glance seems contradictory. However, the two tests measure interactive behaviors under different conditions. In the tube test the two mice are positioned into the ends of a narrow tube and allowed to move forward into the tube to meet; typically, one of the nose-to-nose mice (“the winner”) pushes the other back out of the tube. The resident intruder test takes place in the home cage of the resident and the intruder is introduced. Both mice have access to the whole cage and therefore have the option of avoiding each other. We observed a robust decrease in attacks in IMI-treated resident mice compared to DMSO-treated resident mice, inferring a reduction in aggressive behavior. A similar reduction in attacks and reduced aggressive behaviors in the resident intruder test has been reported for nicotine in several mouse strains including the CD-1 strain used here^52,53^. An alternative interpretation of the data is that the IMI-treated mice displayed a strong propensity to engage in avoidance behavior and/or that the untreated mice engaged in avoidance behavior by reducing contact with the IMI-treated mice. This explanation is compatible with the finding that IMI-treated animals displayed highly significant reductions on several parameters including the number of attacks by the resident, the duration of the attacks, and the total fight time (Fig. 5 and Supplementary Fig. 4). It is conceivable that the reduced aggression and/or avoidance behavior observed may have been asserted through non-visible means, for example by olfactory and/or ultrasonic vocalization cues propagated by the IMI-treated mouse.

Previous work in mouse neuronal cultures has shown that chronic treatment with nicotine induced an up-regulation of [^3^H]epibatidine binding^38^. In the present study, results of the nicotinic receptor binding experiments showed that nicotinic radioligand [^3^H]epibatidine was not different in IMI-treated vs. DMSO-treated mice in the thalamus, cerebral cortex, or superior colliculus; however elevated [^3^H]epibatidine was observed in the interpeduncular nucleus. Increased [^3^H]epibatidine binding could have been caused by residual IMI remaining in this brain region several weeks after treatment, or by an increase in nAChRs induced earlier during peak IMI tissue concentrations, which then subsequently remained elevated in the absence of IMI.

The interpeduncular nucleus is a brain region expressing a very high density of nAChRs^38,40^. The interpeduncular nucleus is thought to have broad inhibitory effects on many other brain regions and is linked with decreased dopamine release and utilization from dopamine producing regions and appears to have a role in the regulation of rapid eye movement sleep. Activation of the GAD2 expressing sub-population of GABAergic inhibitory neurons in the interpeduncular nucleus produces the physical symptoms of nicotine withdrawal suggesting that neurons in this region may be involved in nicotine withdrawal^38^.

Taken together, our findings demonstrate that maternal IMI exposure in mice can be transferred to offspring over the prenatal and early postnatal period and induce perturbations in the behaviors of her adult offspring. Overall, the profile of altered behaviors observed with IMI coincides with those reported for nicotine. Our data suggest that transient exposure to a neonicotinoid insecticide during the critical early developmental period may have potentially long-term consequences that become apparent later in life. It is conceivable that the effects seen might be specific to CD-1 mice, and therefore the effects of IMI should be studied in another mouse strain, and ideally in another mammalian species. Future experimentation should also seek to establish the lowest brain concentration of IMI (and other neonicotinoids) that cause abnormal behaviors. Ultimately, the determination of the lowest tissue concentrations of neonicotinoids that are capable of inducing altered physiology and behavior in laboratory animals, will allow comparisons with the concentrations in food, drinking water, and human tissues. Documented evidence for neonicotinoid bioaccumulation in non-insect invertebrates^54^ and in humans^43^, and the identification of active metabolites of IMI and other neonicotinoids^55–60^ adds further incentive for conducting such studies.

## Electronic supplementary material

Supplementary Information

## Data Availability

The datasets are available from the corresponding author on reasonable request.
