# Supplementary material for: Mammalian Susceptibility to a Neonicotinoid Insecticide after Fetal and Early Postnatal Exposure
Source: Sci Rep. 2018 Nov 9;8:16639. doi: 10.1038/s41598-018-35129-5 (PMC6226530; doi:10.1038/s41598-018-35129-5)
Supplement: Supplementary file 1 — Supplementary Information [file 41598_2018_35129_MOESM1_ESM.pdf]

Supplementary Information

Mammalian Susceptibility to a Neonicotinoid Insecticide after Fetal and Early Postnatal Exposure

Andrew P. Burke, Yosuke Niibori, Hayato Terayama, Masatoshi Ito, Charlotte Pidgeon,

Jason Arsenault, Pablo R. Camarero, Carolyn L. Cummins, Rafael Mateo, Kou Sakabe,

and David R. Hampson

Supplementary Table 1. Overview of behavioural testing. YES indicates that the test was included in the study, while NO indicates that the test was not included in the study.

Tests Conducted	Study A	Study B	Study C
**Elevated Plus Maze**	YES	YES	NO
**Open Field Test**	YES	YES	NO
**Forced Swim Test**	YES	YES	NO
**Tube Test**	YES	YES	YES
**Resident Intruder**	NO	YES	YES

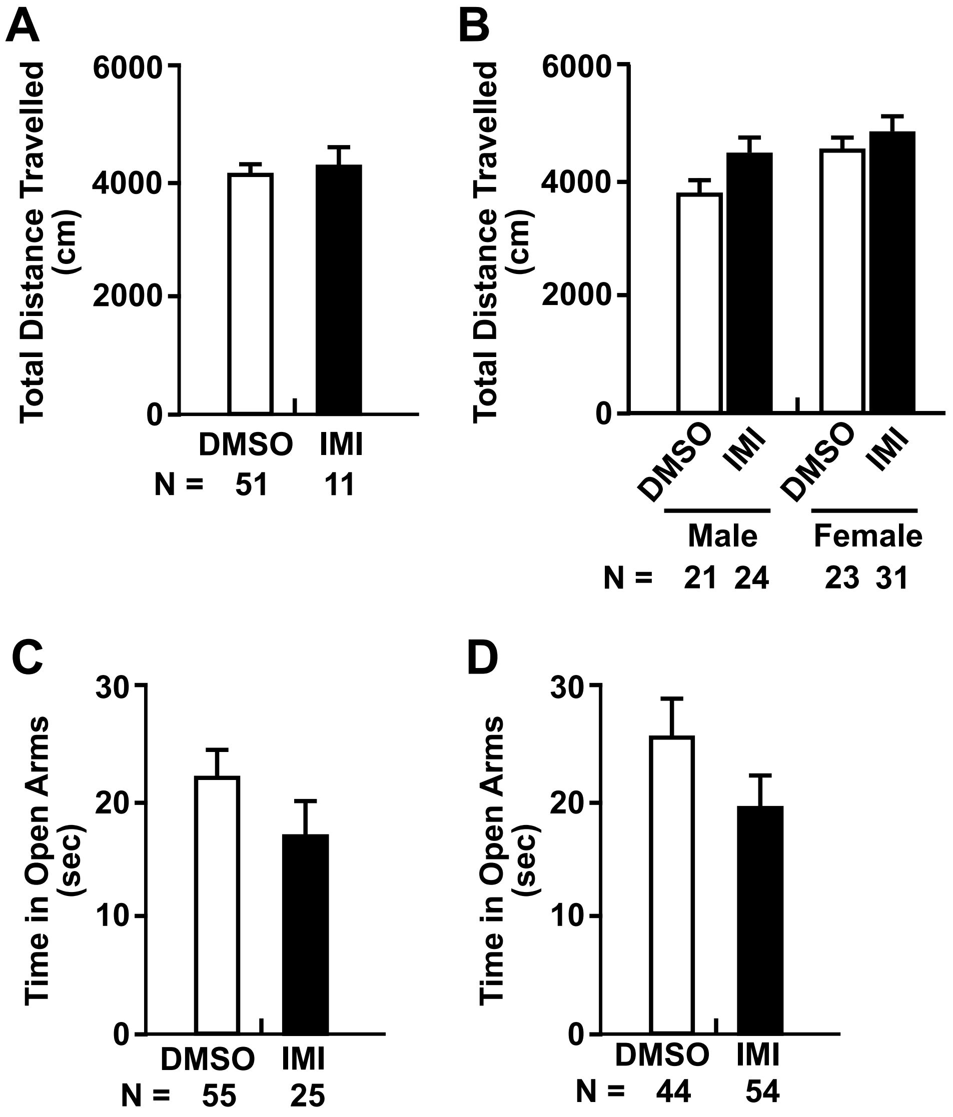

Supplementary Figure 1.
(A) Motor activity analysis from Study A; **(B)** Motor activity analysis from Study B; **(C)** Elevated plus maze, open arm time from Study A; **(D)** Elevated plus maze, open arm time from Study B. In all panels, each column represents the mean ± SEM. N = number of mice in each group. Two-way ANOVA with Bonferroni post-hoc test (A and B), and two-tailed Student's t-test (C and D). $*p < 0.05$.

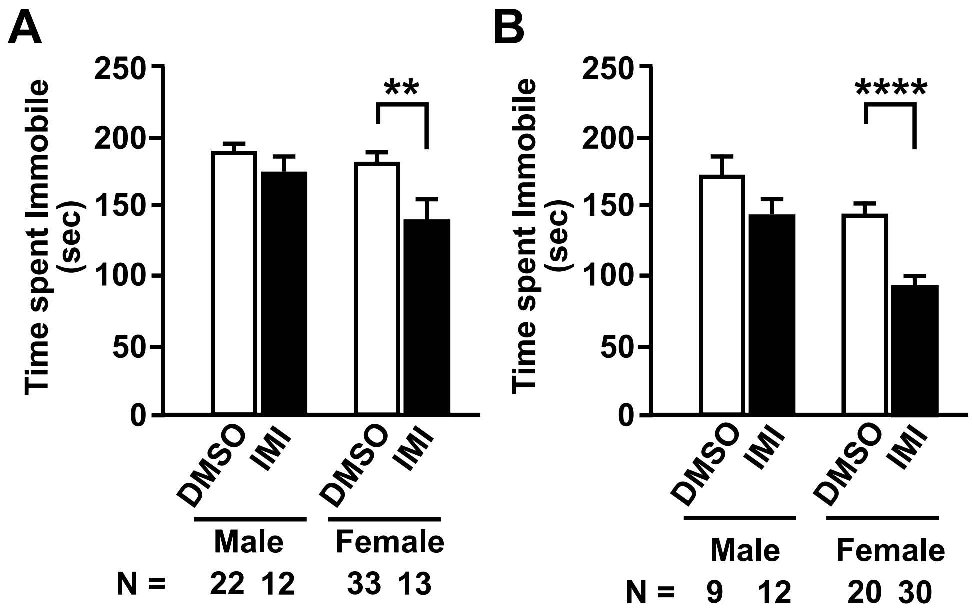

Supplementary Figure 2. Results of the forced swim test. (A) Results of the forced swim test from Study A. **(B)** swim test results from Study B. Each column represents the mean ± S.E.M. N = number of mice in each group. Two-way ANOVA with Bonferroni Post-hoc test. *$p < 0.05$; **$p < 0.01$; ****$p < 0.0001$.

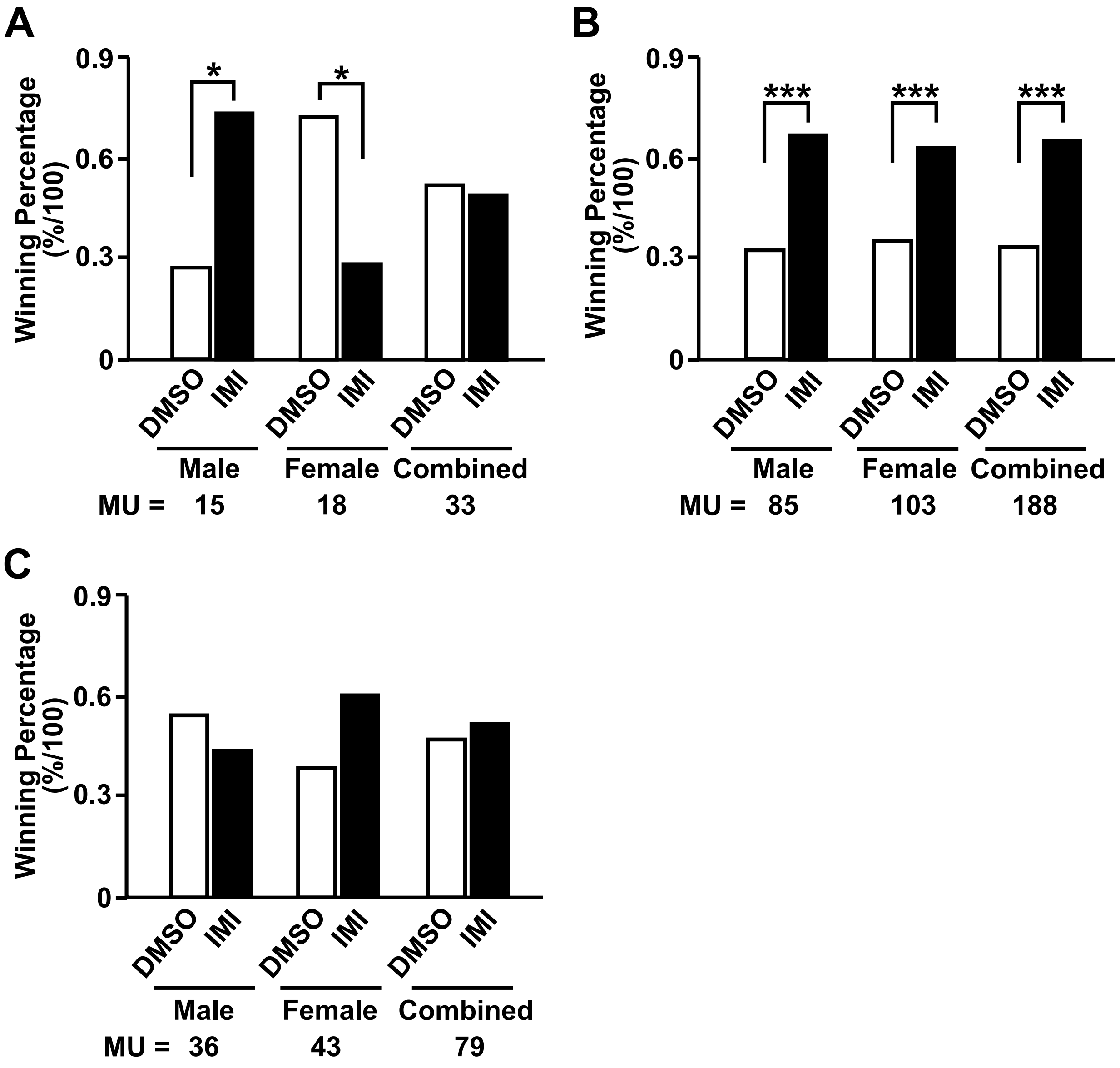

Supplementary Figure 3. (A) Tube test results from Study A; **(B)** Tube test analysis from Study B; **(C)** Tube test results from Study C. No mice were used in more than 5 matchups. The data are presented as the winning percentage, calculated as the number of wins divided by the total number of matchups. MU = number of matchups between the treatment groups. Fisher's exact test, *p < 0.05, ***p < 0.001, ****p < 0.0001.

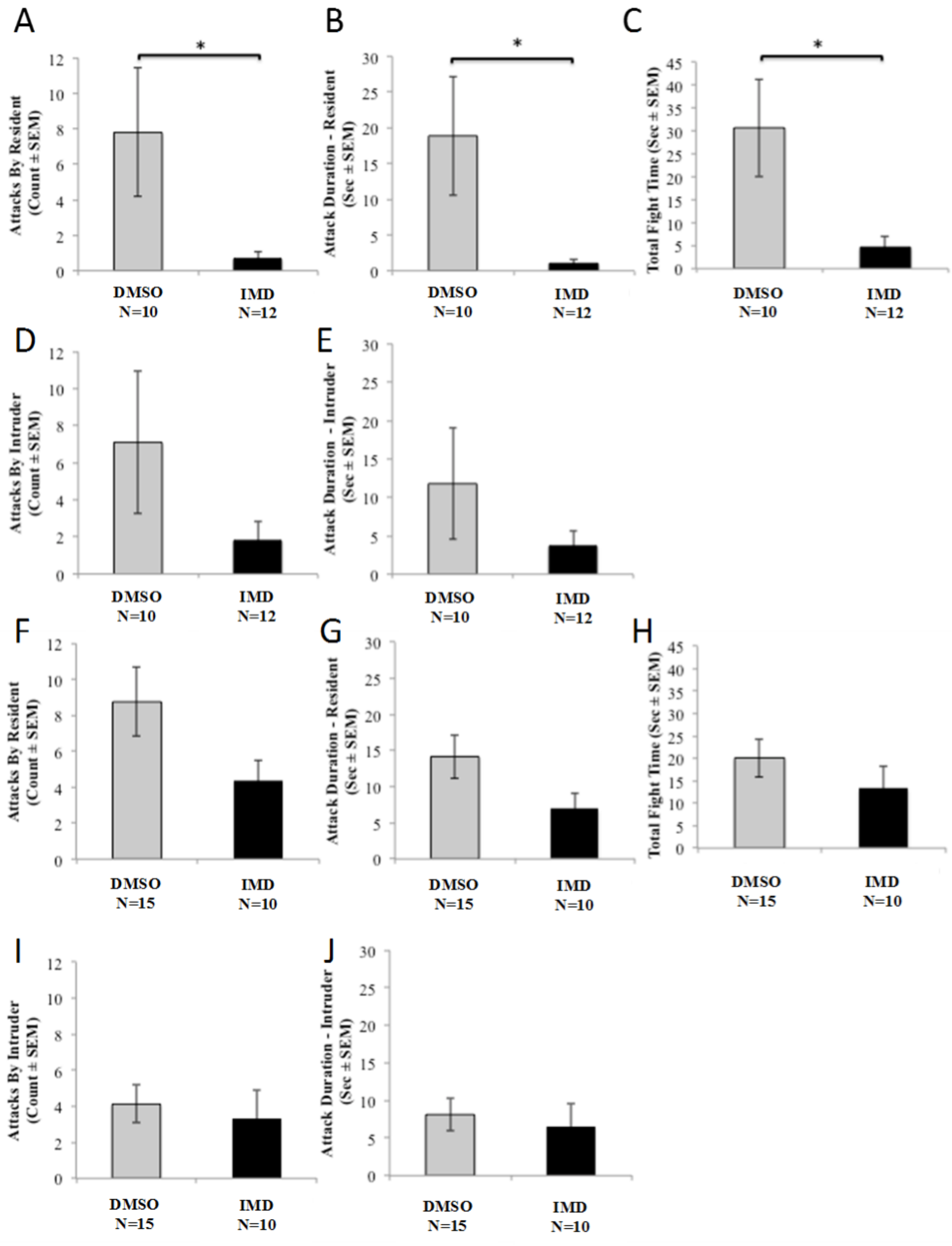

Supplementary Figure 4. Summary of resident intruder test. (A-E) Resident Intruder analysis from Study B. **(F-J)** Resident Intruder analysis from Study C. Each column represents the mean ± SEM. N = number of mice in each group. Two-tailed Student's t-test, *$p < 0.05$.
